# Lipid Delivery Systems for Nucleic-Acid-Based-Drugs: From Production to Clinical Applications

**DOI:** 10.3390/pharmaceutics11080360

**Published:** 2019-07-24

**Authors:** Anna Angela Barba, Sabrina Bochicchio, Annalisa Dalmoro, Gaetano Lamberti

**Affiliations:** 1 Eng4Life Srl, Spin-off Accademico, Via Fiorentino, 32, 83100 Avellino, Italy; 2 Dipartimento di Farmacia; Università degli Studi di Salerno, via Giovanni Paolo II, 132 84084 Fisciano (SA), Italy; 3 Dipartimento di Ingegneria Industriale; Università degli Studi di Salerno, via Giovanni Paolo II, 132 84084 Fisciano (SA), Italy

**Keywords:** NABDs, siRNA, liposomes, clinical trials

## Abstract

In the last years the rapid development of Nucleic Acid Based Drugs (NABDs) to be used in gene therapy has had a great impact in the medical field, holding enormous promise, becoming “the latest generation medicine” with the first ever siRNA-lipid based formulation approved by the United States Food and Drug Administration (FDA) for human use, and currently on the market under the trade name Onpattro™. The growth of such powerful biologic therapeutics has gone hand in hand with the progress in delivery systems technology, which is absolutely required to improve their safety and effectiveness. Lipid carrier systems, particularly liposomes, have been proven to be the most suitable vehicles meeting NABDs requirements in the medical healthcare framework, limiting their toxicity, and ensuring their delivery and expression into the target tissues. In this review, after a description of the several kinds of liposomes structures and formulations used for in vitro or in vivo NABDs delivery, the broad range of siRNA-liposomes production techniques are discussed in the light of the latest technological progresses. Then, the current status of siRNA-lipid delivery systems in clinical trials is addressed, offering an updated overview on the clinical goals and the next challenges of this new class of therapeutics which will soon replace traditional drugs.

## 1. Introduction

Gene therapy is the treatment of diseases at molecular level by “switching genes on or off” through the use of Nucleic Acid Based Drugs (NABDs) which, including oligodeoxynucleotides, plasmid DNA, ribozymes, siRNA, miRNA and related chemically-synthesized molecules, are highly degradable therapeutics that need to be loaded into a vehicle in order to reach the patient target cell/tissue where the defective or mutated gene has to be corrected [1,2]. Indeed, naked genetic materials cannot be easily systemically administered due to their toxicity, low stability in serum, rapid renal clearance, reduced uptake by target cells, phagocyte uptake and their ability in activating the immune response, all features that preclude their clinical development [3]. Therefore, choosing the right vector represents one the most critical steps in attaining a successful gene therapy, and carriers based on lipid materials seem to be the most promising, as they have similar structures to cell membranes [4]. To date, lipid-based NABDs delivery systems represent a novel approach for the treatment of different diseases, mostly including advanced cancers, amyloidosis, fibrosis, hypercholesterolemia and various virus infections, as evidenced by the numerous products under clinical trials, ongoing or planned [3,5,6,7].

**Figure 1:**
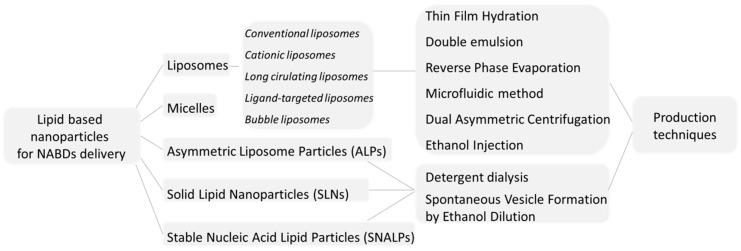
Schematization of lipid-based nanoparticles that are used for Nucleic Acid Based Drugs (NABDs) delivery and their production techniques.

One of the most significant advances in lipid-based NABDs therapies dates back only to August 2018, and is undoubtedly represented by Patisiran (ALN-TTR02), the first ever approved RNAi therapeutic by the Food and Drug Administration (FDA) and by the European Commission (EC). Produced by Alnylam Pharmaceuticals Corporation and sold under the trade name Onpattro™, ALN-TTR02 is an siRNA formulation based upon Stable Nucleic Acid Lipid Particle (SNALP) transfecting technology, and is indicated for the treatment of the Hereditary Transthyretin-mediated Amyloidosis (hATTR), a progressively incapacitating and often fatal genetic disorder. Thus, after 20 years from the first publication about the RNAi-mechanism discovery [8], in August 2018 the gene-silencing technology has had its first drug approval as stated in an article published by Nature and just entitled “Gene-silencing technology gets first drug approval after 20-year wait” [9]. It appears evident how the clinical translation of lipid-based NABDs delivery systems shelf has progressed [10,11,12,13,14,15,16,17], leading to the formulation of several lipid-based carriers (see schematization of Figure 1), including micelles, solid lipid nanoparticles and liposomes [18].

Among these systems, liposomes are the most effective system. They are spherical particles composed of phospholipids, encapsulating a volume of aqueous medium showing several favorable features, such as an excellent biocompatibility, biodegradability, low toxicity, low immunogenicity, an ability to deliver a large piece of nucleic acids, structural flexibility and easiness of handling. Moreover, cationic liposomes, made by positively-charged lipids, are especially indicated for NABDs delivery, being this last characterized by negatively-charged phosphate groups. Their association gives compacted structures which can also electrostatically interact with the negatively-charged cell membrane, facilitating their cellular uptake [19]. In particular, in recent years, cationic liposomes have gained a lot of attention for siRNA delivery, improving their uptake into tumor tissues and their stability and bioavailability [20,21]. The siRNAs are 21–23 nucleotides long, double-stranded RNA, which, binding at specific sequences of messenger RNA (mRNA), “interfere” with the translation of proteins. Their therapeutic potential as a next generation medicine has been recently reviewed [22], together with the major delivery strategies currently adopted for their effective in vitro and in vivo delivery [23].

In this work an overview on the advances in NABDs-lipid systems delivery is presented. In particular, the attention is before focused upon liposomes as a lipid carrier system and on siRNA as NABDs. After, a discussion about liposomes structures, the formulations and production techniques suitable for NABDs delivery are reported. In the last part, the medical applications of siRNA-liposomes currently under investigation in clinical trials are then treated in detail.

## 2. Liposomes: Structures and Basic Formulations

Liposomes are vesicles characterized by a lipid bilayer that surrounds an aqueous core. They are mainly composed of phospholipids recognizable by a polar hydrophilic head and two apolar hydrophobic chains [24]. 

**Figure 2:**
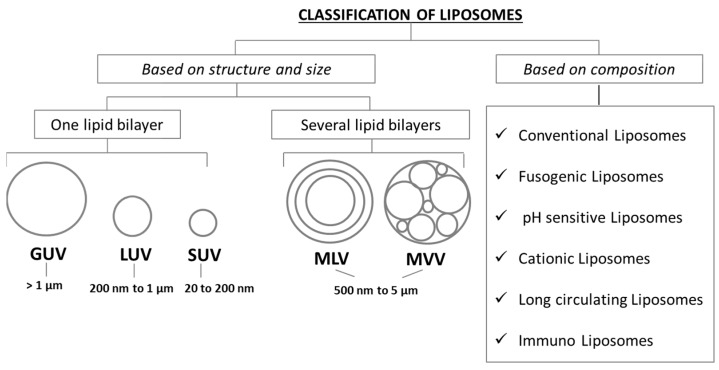
Liposomes classification by structure/size and lipid composition.

When dispersed in aqueous solutions, their polar heads interact with the aqueous environment, due to the hydrogen bonds and polar interactions, while their aliphatic chains interact with each other due to the van der Waals forces, leading to a lipid bilayers formation of which they constitute the lipophilic inner compartment [24,25]. During their formulation process, water-soluble drugs can be dissolved in the aqueous compartment while hydrophobic materials can be entrapped into the lipid bilayer [26,27,28]. Joined by being composed of biocompatible materials, liposomes show different structures, dimensions, lipid composition and surface charge. They can be composed by several concentric bilayers separated by aqueous compartments, with an external lipid bilayer containing other ever smaller bilayers separated by water cavities, like an onion structure. In this case liposomes are called Multilamellar Vesicles (MLVs) and show a size range of 500 nm to 5 µm, or by only one phospholipid bilayer surrounding an aqueous compartment. In this case liposomes can be differentiated in small, large and giant vesicles depending upon their dimension: They are called Small Unilamellar Vesicles (SUVs) if they have a 20 to 200 nm range size, Large Unilamellar Vesicles (LUVs) with a 200 to 1 µm range size and Giant Unilamellar Vesicles (GUVs) with a size larger than 1 µm. Finally, similar in dimension to MLVs there are multi-compartmental structures constituted by vesicles surrounded by other vesicles called Multi Vesicular Vesicles (MVVs) [19,29,30]. In Figure 2 a schematization of liposomes classification based on their structure, size and composition is presented.

Liposomes are mainly made of phospholipids, which contain two major categories including glycerophospholipids and sphingomyelins. Composed by a hydrophilic head group, a hydrophobic side chain and a glycerol backbone, glycerophospholipids are those that characterized the eukaryotic cells, and are used in liposomes production by varying their head group i.e., phosphatidylcholine (PC), phosphatidylethanolamine (PE), phosphatidylserine (PS), phosphatidylglycerol (PG) [31].

### 2.1. Conventional Liposomes

Conventional liposomes are neutrally-charged vesicles made primarily from PC and cholesterol; the incorporation of this last into the lipid bilayer, with its hydroxyl group oriented towards the aqueous surface, and the aliphatic chain aligned parallel to the acyl chains [2], enhances the stability of the vesicles, reducing their permeability, and increasing their in vivo and in vitro performance [24,32,33,34].

Although reducing the in vivo drugs toxicity, being recognized as foreign substances by serum opsonines and destroyed by the reticuloendothelial system (RES), conventional liposomes are characterized by a short circulation time when intravenously administered [17,35]. 

To overcome this limit, the strategy of a vesicle surface modification by hydrophilic inert polymers has been adopted for the development of stealth liposomes, and the approach is discussed in the “Long circulating liposomes” paragraph. Moreover, the use of conventional liposomes for NABDs delivery usually results in a scarce encapsulation efficiency due to the lack of electrostatic interactions between the positive nucleic acids and the uncharged vesicles bilayer. To avoid this problem, cationic liposomes can be used, as discussed below.

### 2.2. Cationic Liposomes

Vesicles can be prepared by using positively-charged lipids, i.e., the 1,2-DiOleoyl-3-TrimethylAmmonium Propane (DOTAP), achieving cationic liposomes which can electrostatically attract the negative phosphate groups of NABDs, increasing their encapsulation efficiency. Moreover, the cationic liposomes also facilitate the intracellular uptake of NABDs and their endosomal escape, more efficiently if “helper” lipids are included in the formulation [19].

**Figure 3:**
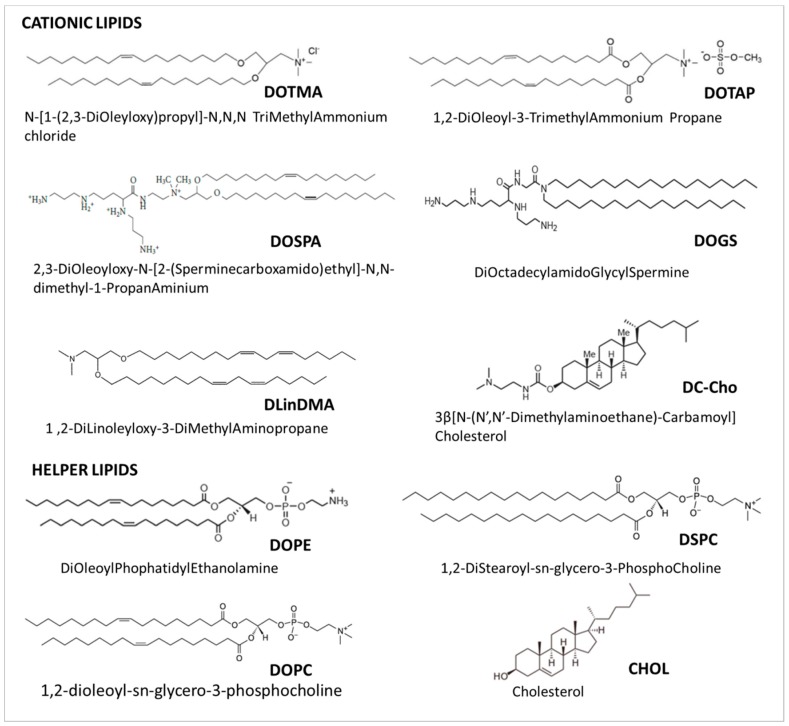
Structures of cationic and helper lipids used as components of various siRNA liposomal delivery systems.

Indeed, neutral and zwitterionic lipids, mainly 1,2-dioleoylsn-glycero-3-phosphatidylcholine (DOPC), Di-Oleoyl-Phosphatidyl-Ethanoalamine (DOPE) and 1,2-DiStearoyl-sn-glycero-3-PhosphoCholine (DSPC), being more fusogenic than the cationic one, can affect the polymorphic features of the liposome–siRNA complexes, promoting the transition from a lamellar to a hexagonal phase, thus inducing fusion and a disruption of the membrane [36]. In particular the fusogenicity increases with the decrease of the degree of the saturation of the lipids hydrophobic tails, and with the decrease of their polar heads size (i.e., lipids with large polar heads and highly saturated tails prefer the lamellar phase, and thus are not fusogenic; lipids with small polar heads and highly unsaturated tails prefer the reverse hexagonal phase, and thus are fusogenic) [15]. DOPC lipid has been successfully used by the M.D. Anderson Cancer Center to produce siRNA-EphA2-DOPC, which is currently in a Phase I clinical trial for the treatment of advanced cancers, as discussed later in the “Current applications of lipid-based siRNA delivery” Section. Some of the most common cationic and helper lipids used for NABDs delivery are reported in Figure 3.

Cationic liposomes formulation is in constant improvement. In order to optimize siRNA encapsulation efficiency, Santel and collaborators have synthesized the cationic lipid β-l-Arginyl-2,3-l-diaminopropionic acid-N-palmityl-N-oleyl-amide trihydrochloride (AtuFECT), which when complexed with siRNA is termed AtuPLEX [37]. Atu027 is an AtuPLEX produced to treat advanced solid tumor [38] which has completed the Phase I and the Phase I/II of clinical trials (see Section 4).

Cationic liposomes can be also formulated by adding to the basic vesicles composition the lipidoids, lipid-like materials produced through the conjugate addition of an amine to an acrylate or acrylamide [4,39,40,41,42,43]. Akinc and collaborators have produced a complex called “LNP01” which is made by 98N12-5:cholesterol:PEG-lipid in a molar ratio of 42:48:10 (mol:mol:mol) respectively, with a total lipid:siRNA ratio of about 7.5:1 (wt:wt), a C14 alkyl chain length on the PEG lipid and a mean particle size of roughly 50–60 nm. The developed formulation is liver targeted (with a >90% injected dose distributed to the liver) and can induce, after repeated administrations, a long-duration gene silencing without any loss of activity [42,43].

**Figure 4:**
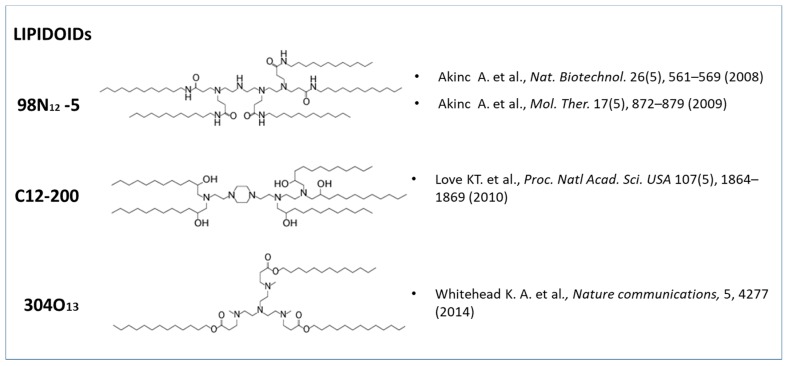
Lipidoids synthetized by different research groups and used in cationic liposomal formulations to increase siRNA encapsulation efficiency.

Love and coworkers have also made complexes by combining lipidoid, cholesterol, and a polyethylene glycol modified lipid in a yield weight fractions of 52:20:28 (wt:wt:wt), obtaining an siRNA-directed liver gene silencing in mice at doses below 0.01 mg/kg [41]. A lipidoid nanoparticle which mediates potent gene knockdown in hepatocytes and immune cell populations after IV administration to mice, with siRNA EC50 values as low as 0.01 mg/kg, was successfully produced also by Whitehead and collaborator [39]. The lipidoids structures used in the cited works for siRNA-lipid complexes production are given in Figure 4.

Finally, several commercial products based on cationic liposomes for NABDs transfection during in vitro experiments are available on the market, the most used ones are Lipofectine and LipofectAMINE, produced and sold by the Invitrogen Company.

Despite the advantages in strongly attracting NABDs, the use of cationic liposomes presents several drawbacks due to their instability, rapid systemic clearance, toxicity and their induction of immunostimulatory responses [44,45]. Alternatively, in order to ameliorate cationic liposomes performances in gene therapy, different techniques were developed for the production of relatively new cationic lipid structures, including Stable Nucleic Acid Lipid Particles (SNALP), as discussed in Section 3.

### 2.3. Long Circulating Liposomes

The rapid decrease of liposomal drug complexes in blood with the consequent accumulation in liver, spleen and other organs has led to the development of the long circulating liposomes, lipid vesicles characterized by a surface covered with inert polymeric molecules, such as oligosaccharides, glycoproteins, polysaccharides and synthetic polymers [28]. Among these, covering liposomes with the polyethylene glycol (PEG) represents an effective strategy to increase the repulsive forces between liposomes and serum-components, thus avoiding their elimination by the RES, while improving their stability and enhancing their circulation times in the blood [46,47,48]. Such surface modified liposomes have been called “PEGylated” or “stealth” liposomes, since they can evade recognition by T cells and macrophages, and avoid rapid clearance by the immune system [49,50]. In particular, the polymeric chain adsorbed on the liposomes surface consisting of tails, loops and trains, can form several structures on the bases of the polymeric layer size formed. Polymeric “pancake”, “mushroom” or “brush” structures are formed with a PEG surface thickness of about 1.5 nm, 3.5 nm and 5 nm, respectively [18,51]. Doxil^®^ represents the first FDA-approved drug carrier based on PEGylated liposome technology for the treatment of advanced ovarian cancer, multiple myeloma and HIV-associated Kaposi’s sarcoma [52]. Moreover, several PEGylated liposomal products for NABDs delivery are in preclinical development, i.e., the lipidoid-based siRNA formulation 98N12-5 [53] or the DACC cationic lipoplex [54].

However, the long-circulating liposomes exhibit some drawbacks: (1) The PEGylation has to be optimized by modulating PEG length and density in order to control the interactions between the liposomal, the plasmatic and the endosomal membranes, which can prevent the vesicles’ cellular uptake/escape from the endosome. This problem, known as “PEG dilemma”, can be avoided by using cleavable PEG-lipids such as the “pH-responsive” or “enzyme-responsive” ones [47]; (2) covering with PEG does not guarantee the selectivity of the drug delivery to the target tissue. This problem can be overcome by using the targeting ligands conjugation approach [55] discussed below.

### 2.4. Ligand-Targeted Liposomes

Tumor tissues/blood vessels are characterized by specific and overexpressed receptors which can bind to antibodies, polysaccharides, proteins, polypeptides, aptamers and other molecules. The attachment of these specific targets to the NABDs-liposomes surface guarantees an active targeting of the carriers to the diseased tissue [28]. Recently, a Dual-Targeting Ligands approach was developed by Riaz and collaborators [49], and a combination of peptide and antibody ligands was used to fuctionalize a single liposomal formulation, obtaining an increased nanoparticles cell uptake [56].

In 1995 folic acid was conjugated for the first time onto the surface of nucleic acids liposomal carriers (folate-PEG-liposomes) by Wang and collaborators [57]. To date, the folate receptor is one of the most used targets for siRNA liposomal delivery, being overexpressed in several malignancies, such as ovarian and uterine cancer, osteosarcoma, meningioma and other cancer cells, but not in normal tissues [28].

The transferrin receptor-dependent mechanism is also used for liposomal NABDs internalization, i.e., Mendoça and coworkers have successfully developed a transferrin receptor-targeted liposome loaded with an anti-BCR-ABL siRNA or asODN for the treatment of chronic myeloid leukemia [58].

Recently, Zang and collaborators have developed a pH-sensitive cholesterol-Schiff base-polyethylene glycol (Chol-SIB-PEG)-modified cationic liposome–siRNA complex, conjugated with the recombinant humanized anti-EphA10 antibody (Eph). The Eph–PEG–SIB–Chol-modified liposome–siRNA complex (EPSLR) has shown a good endo-lysosomal escape, releasing siRNA into the cytoplasm after 4 h from in vitro transfection. Moreover, an in vivo study conducted in tumor-bearing mice shows that EPSLR can reach the diseased tissue more effectively [59].

Moreover, polysaccharides, i.e., galactose, mannose, dextran and hyaluronic acid can be also used as ligands to prepare glycosyl-liposomes targeting tissues overexpressing these receptors. The use of functionalization with polysaccharides and other targeting moieties for an effective NABDs delivery has been widely described in [18].

### 2.5. Bubble Liposomes

Lipid vesicles filled with gas are called “Bubble Liposomes”. They are particles ranging from 1 to 10 micro meters, characterized by a gas core and a shell composed of several materials i.e., proteins, lipids, polymers, surfactants and galactose, used to deliver NABDs and other drugs into the cells and tissues [60]. In particular, the term “Bubble Liposomes” was coined by Suzuki, Maruyama and collaborators, who developed novel liposomes containing lipid nanobubbles loaded with perfluoropropane, used as ultrasound imaging agent. Briefly, “Bubble Liposomes” are prepared by producing at first polyethyleneglycol-modified liposomes (PEG-liposomes) through the reverse phase evaporation method, and placing them in vials with perfluoropropane gas, then sonicating in a bath sonicator. Upon exposure to ultrasound, these particles can induce cavitation, which supplies the energy required to deliver extracellular molecules into the cytosol, and this can thus be utilized as a gene delivery tool [61]. This phenomenon involves a greater cell/tissue permeability, improving NABDs penetration into the cells and ensuring, at the same time, their escape from the endosome with their consequent expression [62].

In that regard, it was demonstrated that bubble liposomes can deliver genes into cells even when the cells were exposed to ultrasound for only 1 s. They have also shown that this kind of liposome is more effective in delivering NABDs, i.e., the luciferase gene, into a tumor than are the conventional cationic liposomes used as transfection agents [63].

Recently, several research groups have opted for the use of bubble liposomes for NABDs delivery such as Negishi and collaborators, who have used bubble liposomes for a selective gene delivery to syndecan-2 overexpressing cancer cells [64], or also Endo-Takahashi and coworkers, in whose work bubble liposomes were successfully used to deliver miRNA (miR-126) for the cure of hindlimb ischemia by the systemic administration of miR-126-loaded bubble liposomes into mice coupled with US exposure [65].

## 3. Liposomes Preparation Techniques

There are many different methods for the preparation of liposomes [19,66,67,68], here the attention is focused on those available for NABDs encapsulation.

### 3.1. Thin Film Hydration

The most commonly used technique for liposomes preparation is the Thin Film Hydration (TFH) or the Bangham method, in which lipids are dissolved in an organic solvent, then evaporated through the use of a rotary evaporator leading to a thin lipid layer formation [69]. After the layer hydration by an aqueous buffer solution containing the hydrophilic drug to be loaded, Multilamellar Vesicles (MLVs) are formed, which can be reduced in size to produce Small or Large Unilamellar vesicles (LUV and SUV) by extrusion through membranes or by the sonication of the starting MLV [18,19,70]. Even if the method is not scalable, and toxic solvent traces could remain in the final formulation, its easiness to perform and low cost make this one of the most adopted techniques for liposomes preparation. The method is used also for the production of liposomes containing NABDs, whose encapsulation efficiency, however, is generally quite low, ranging from 3 to 45%, but can be increased by modulating the lipid mixtures [71,72]. In our previous works DOTAP cationic lipid was added to a cholesterol and phosphatidylcholine formulation in order to electrostatically attract siRNA molecules. Briefly, through the TFH method, followed by a duty cycle sonication, siCyD1-nanoliposomes [73] and siE2F1-nanoliposomes [74] with 100% siRNA encapsulation efficiency, able to reduce respectively colon CyD1 and E2F1 proteins expression after transfection in ex vivo human tissue cultures, were produced.

By modulating the lipid formulation adding DOTAP, Coated Cationic Liposomes (CCL) entrapping an antisense oligodeoxynucleotides (asODN), with a diameter of 188 nm and an encapsulation efficiency of 85–95% were also prepared through the TFH technique by Stuart and coworkers [75].

### 3.2. Double Emulsion

Liposomes can be also prepared through the Double Emulsion technique which involves lipids dissolution in a water/organic solvent mixture. The organic solution, containing water droplets, is mixed with an excess of aqueous medium, leading to a water-in-oil-in-water (W/O/W) double emulsion formation. After mechanical vigorous shaking, part of the water droplets collapse, giving Large Unilamellar Vesicles (LUVs) [76]. This method was used for NABDs encapsulation, but achieved a very low entrapment efficiency [77].

### 3.3. Reverse Phase Evaporation

The Reverse Phase Evaporation (REV) method also allows one to achieve LUVs loaded with NABDs [78]. In this technique a two-phase system is formed by phospholipids dissolution in organic solvents and aqueous buffer. The resulting suspension is then sonicated briefly until the mixture becomes a clear one-phase dispersion. The liposome formation is achieved after the organic solvent evaporation under reduced pressure. This technique has been used to encapsulate different large and small hydrophilic molecules included nucleic acids, i.e., in a work of Stuart and Allen, cationic vesicles loaded with nucleic acids and covered with neutral lipids, with significantly high incorporation efficiency (80–100%), and being stable in 50% human plasma at 37 °C, were produced [79]. Recently by means of a modified REV, Mokhtarieh and collaborators have produced Asymmetric Liposome Particles (ALPs) containing siRNA of about 200 nm in size with more than 90% encapsulation efficiency. The method provides the formation of two kinds of lipid inverted micelles composing the inner and outer lipid film. The siRNAs are entrapped in the inner one, made of ionizable cationic lipids, which is mixed with the outer lipid film made of conventional lipids. After a solvent evaporation and dialysis, siRNA-ALPs are achieved [80].

### 3.4. Microfluidic Method

A relatively new technology used for liposomes production is the Microfluidic method, unlike other bulk techniques, this one gives the possibility of controlling the lipid hydration process. The method can be classified in continuous-flow microfluidic and droplet-based microfluidic, according to the way in which the flow is manipulated [81]. In 2004, Jahn and collaborators described a microfluidic hydrodynamic focusing (MHF) method which operates in a continuous flow mode. Briefly lipids are dissolved in isopropyl alcohol which is hydrodynamically focused in a microchannel cross junction between two aqueous buffer streams. Vesicles size can be controlled by modulating the flow rates, thus controlling the lipids solution/buffer dilution process [82]. The method was successfully extended for producing oligonucleotides (ON) lipopolyplexes by using a microfluidic device consisting of three-inlet and one-outlet ports. Lipids ethanol solution and ON aqueous solutions are contained into sterile syringes connected to the inlet ports. Briefly, a fluid stream is split into two side streams at inlet port 1 or 2, while a fluid stream directly entered the center microchannel through inlet port 3. The resulting ON liposomes (about 115 nm in size) solution is collected at the outlet port [81].

### 3.5. Dual Asymmetric Centrifugation

Dual Asymmetric Centrifugation (DAC) is another method for the production of NABDs-liposomes [83]. This technique differs from the usual centrifugation because the sample is undergone, during the normal centrifugation process, to an additional rotation around its own vertical axis. By this way an efficient homogenization is achieved due to the two overlaying movements generated: The sample is pushed outwards, as in a normal centrifuge, and then it is pushed towards the center of the vial due to the additional rotation. Briefly, by mixing lipids and an NaCl-solution a viscous vesicular phospholipid gel (VPC) is achieved, which is then diluted to obtain liposomal dispersion. Liposome size can be regulated by optimizing DAC speed, lipid concentration and homogenization time. With this method Hirsch and coworkers have prepared siRNA-liposomes, about 109 nm in size, with high entrapping efficiency, ranging from 43 to 81%, depending upon batch size [84]. In 2011, also Adrian and collaborators have used the DAC method to produce liposomes containing siRNA, targeting the particles surface with an antibody for the selective interaction with neuroblastoma cells, achieving 190 to 240 nm particles with siRNA encapsulation efficiency of up to 50% [85]. By this technique, in only one step, sterile SUVs formulations in a highly reproducible manner can be prepared [86].

### 3.6. Ethanol Injection

Unilamellar liposomes can be prepared by the Ethanol Injection (EI) method which can be used for NABDs encapsulation. This method provides the rapid injection of an ethanolic solution, in which lipids are dissolved, into an aqueous medium containing nucleic acids to be encapsulated, through the use of a needle. Vesicles are spontaneously formed when the phospholipids are dispersed throughout the medium. By utilizing this method Stabilized Antisense-Lipid Particles (SALPs) can be obtained [87], i.e., Saffari and collaborators showed that by the Ethanol injection method DOTAP-liposomes loaded with an antisense oligonuclotide (AsODN) against protein kinase C alpha, with a size of 115 nm, not requiring downsizing with extrusion or other methods, can be achieved. They obtained an encapsulation efficiency of around 90% that was about seven times more than that of the TFH method, also tried out in their work [88]. In a work of Saad and collaborators cationic liposomes were produced by the EI method for the co-delivery of doxorubicin and siRNA targeted to MRP1 and BCL2 mRNA (suppressors of pump and nonpump cellular resistance) to multi-drug resistance (MDR) lung cancer cells, enhancing the chemotherapy efficiency [89].

### 3.7. Detergent Dialysis

The Detergent dialysis method can be used to encapsulate plasmid DNA. Briefly lipid and plasmid are solubilized in a detergent solution of appropriate ionic strength, after removing the detergent by dialysis, Small Stabilized Plasmid Lipid Particles (SPLPs) are formed. Unencapsulated DNA is then removed by ion-exchange chromatography and empty vesicles by sucrose density gradient centrifugation. The technique is highly sensitive to the cationic lipid content and to the salt concentration of the dialysis buffer, and the method is also difficult to scale [90]. Through this technique 100 nm liposomes loaded with nucleic acids with 60–70% encapsulation efficiency were achieved [91].

### 3.8. Spontaneous Vesicle Formation by Ethanol Dilution

SPLPs can also be produced through the Spontaneous Vesicle Formation by Ethanol Dilution method in which a stepwise or dropwise ethanol dilution provides the instantaneous formation of vesicles loaded with plasmid DNA by the controlled addition of lipid dissolved in ethanol to a rapidly mixing aqueous buffer containing DNA. In a work of Jeffs and collaborators, SPLP prepared by the stepwise approach had a DNA encapsulation efficiency of 81% and were monodisperse with a mean vesicle diameter of about 130 nm, while SPLP prepared by dropwise ethanol had a DNA encapsulation efficiency of 74% and mean vesicle size of about 115 nm [92]. Later the method has been applied successfully to NABDs smaller than plasmids, such as miRNAs and siRNAs, obtaining a Stable Nucleic Acid Lipid Particle (SNALP) of about 100 nm with a high nucleic acids encapsulation efficiency (>95%) [93,94]. In a work of Judje and collaborators 2′OMe-modified siRNA targeting apolipoprotein B (apoB) was successfully encapsulated inside 100 to 130 nm liposomes with an entrapment efficiency of 90–95% through the stepwise ethanol dilution method, obtaining a potent silencing of the endogenous gene target apoB when administered systemically in animals [95]. Recently, Wilner and Levy have described a method for targeting SNALP encapsulating siRNA with aptamers in order to obtain a selective targeted delivery along with an efficient siRNA-mediated gene knockdown [44,96].

Due to their stability, their ability in remain intact in circulation for many hours and accumulate at the target sites, the SNALPs systems have been successfully adopted in a variety of NABDs formulations which are currently in clinical development for the cure of several diseases i.e., solid tumors, Amyloidosis, Hepatitis B, Hypercholesterolemia and the Ebola Virus (see Section 4) [97,98,99,100].

### 3.9. siRNA Encapsulation in Preformed Liposomes

Finally, the entrapment of NABDs can be also obtained starting with preformed liposomes through two different methods: (1) A simple mixing of cationic liposomes with nucleic acids which gives electrostatic complexes called “lipoplexes” [101], where they can be successfully used to transfect cell cultures, but are characterized by their low encapsulation efficiency and poor performance in vivo [72,102]; and (2) a liposomes destabilization, slowly adding absolute ethanol to a cationic vesicles suspension up to a concentration of 40% v/v followed by the dropwise addition of nucleic acids achieving loaded vesicles; however, the two main steps characterizing the encapsulation process are too sensitive, and the particles have to be downsized [72].

## 4. Current Applications of Lipid-Based siRNA Delivery

**Figure 5:**
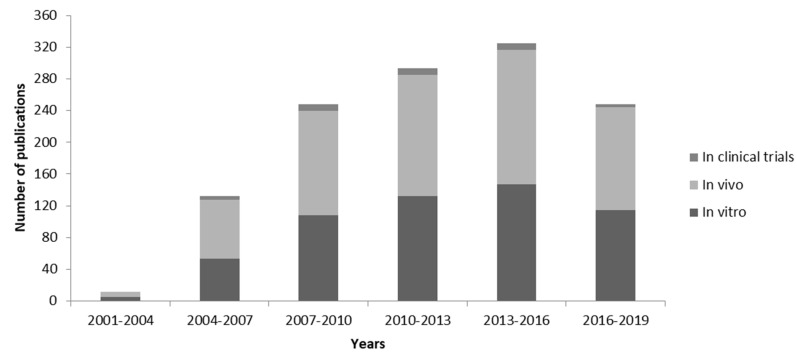
PubMed publications related to “siRNA liposome”. Data recorded in the PubMed database were identified with the search terms “*siRNA liposome*” and “*in vitro”* or “*in vivo”* or “*in clinical trials*” used for the search query. Data were compiled and plotted as a bar graph for the number of publications since 2001 and every three years; data for 2019 are based on the first five months.

During the last 20 years we assisted in a fast growth of the attention of the scientific community relative to the RNAi mechanism, and thus to NABDs new formulations, including small interfering RNA (siRNA), associated with lipid-based delivery strategy to be use for the cure of several diseases. As illustrated in Figure 5, starting from the 2001, year in which siRNA was successfully delivered into mammalian cells for the first time [103], until 2019, the numbers of publications related to “siRNA and liposome” in vitro, in vivo and in clinical trials applications, have increased dramatically, and different liposomal-based siRNA formulations have already shown good safety records in humans.

In particular, here the attention is focused on the applications of siRNAs delivered by lipid-based systems currently into the clinical trials stage. The clinical trials are summarized in Table 1, reporting the common name of the drug, the gene targeted, the disease which it is intended to cure, the company which is carrying out the experimentation, the identifier, the phase (phase I: Pharmacodynamics and Pharmacokinetics—sometimes noted as phase 0 and screening for safety; phase II: Establishing the efficacy of the drug; phase III: Final confirmation of safety and efficacy) and its status as obtained from [104]. In all the clinical trials, the pharmaceuticals were administered by intravenous injection (bolus and/or infusions), and the delivery system is a lipid-based one.

Alnylam Pharmaceuticals is the most active company in the field of siRNA therapeutics. Some of its drugs were delivered by the Stable Nucleic Acid Lipid Particles (SNALPs) technology of the Tekmira Pharmaceuticals Corporation. Several pharmaceuticals using this and other lipid-based delivery technologies involved in the clinical trials are listed below.

1. ALN-VSP02

The ALN-VSP02 is an Alnylam Pharmaceutics lipid nanoparticle formulation containing two small interfering RNAs (siRNAs) directed against the kinesin spindle protein (KSP) and vascular endothelial growth factor (VEGF) mRNAs, preventing their translation. Since their key role in tumor proliferation, VEGF and KSP downregulation can lead to a growth inhibition of the cells implicated in such disease. ALN-VSP02, produced through the Tekmira’s SNALP technology, entered in a phase I dose-escalation trial (NCT 00882180, 2009, “A Multi-Center, Open Label, Phase 1 Dose-Escalation Trial to Evaluate the Safety, Tolerability, Pharmacokinetics and Pharmacodynamics of Intravenous ALN-VSP02 in Patients with Advanced Solid Tumors with Liver Involvement”). The study was completed, and a total of 41 patients were enrolled between Mar 2009 and Aug 2011, including 30 patients of the dose-escalation phase treated at 0.1 to 1.5 mg/kg (administered by IV infusion) and 11 of the expansion phase treated at 1.0 or 1.25 mg/kg. On the basis of the molecular analysis of biopsy samples from patients, the study confirmed the presence of residual siRNAs and mRNAs cleavage products at the site of actions. The drug was well tolerated at the highest dose, and then an expansion study has been initiated and completed (NCT01158079, 2010, “A Multi-center, Open-Label, Extension Study of ALN-VSP02 in Cancer Patients Who Have Responded to ALN-VSP02 Treatment”). Seven patients from the first study were treated for an average of 11.3 months (between Jul 2010 and Aug 2012), in order to further evaluate the safety, tolerability, pharmacokinetics and pharmacodynamics of the drug. Even if the efficacy of the drug was not tested during the phase I clinical trial, it is worth noting that one patient (of seven) with endometrial cancer shown a complete response (full tumor regression) and he has remained in remission and completed treatment after receiving 50 doses over 26 months. Three other patients have shown stable disease, having received 17–36 doses over 8–18 months. The results of these two studies are reported in [105].

2. ALN-PCS02

The ALN-PCS02 is a SNALP-formulated [106] RNAi therapeutic targeting PCSK9 mRNA, encoding for Convertase Subtilisin/Kexin type 9 (PCSK9) protein for the treatment of hypercholesterolemia. Binding to Low-Density Lipoprotein (LDL) receptors, PCSK9 leads to their degradation. Genetics studies have shown that reduced-plasma LDL cholesterol can be noticed as a consequence of the loss-of-function mutations in PCSK9 resulting in a decreased risk of coronary heart disease. Therefore, drugs that block PCSK9 can lower LDL. 

Preclinical studies have given encouraging results in mice [107], thus a phase I clinical trial was started by Alnylam Pharmaceutics (NCT01437059, 2011, “A Phase 1, Randomized, Single-blind, Placebo-Controlled, Single Ascending Dose, Safety, Tolerability and Pharmacokinetics Study of ALN-PCS02 in Subjects With Elevated LDL-Cholesterol (LDL-C)”). The study, started in Sep 2011 and ended in Mar 2012, enrolled 32 patients with elevated LDL-Cholesterol levels. 24 over 32 patients received the siRNA-SNAPL pharmaceutical, dosed between 0.015 and 0.400 mg/kg, noticing an adverse effect (AE) level equal to that of the control group (eight patients treated with a placebo). Finally patients who received ALN-PCS at 0.400 mg/kg showed a 70% reduction in circulating PCSK9 plasma and a mean of 40% reduction in LDL-C with respect to those treated with the placebo [108].

3. ALN-TTR01 and ALN-TTR02

Two distinct siRNA-SNAPL formulations, the ALN-TTR01 and ALN-TTR02, targeting the TransThyRetin (TTR, TRANsports THYroxine and Retinol) gene encoding for the protein TransThyRetin (TTR), were developed by Alnylam Pharmaceuticals for the cure of amyloid diseases associated with a TTR overexpression. Both of the formulations have been investigated in phase I clinical trials (NCT 01148953, 2010, “A Phase 1, Randomized, Single-Blind, Placebo-Controlled, Dose Escalation Trial to Evaluate the Safety and Tolerability of a Single Dose of Intravenous ALN-TTR01 in Patients With TTR Amyloidosis”) and (NCT 01559077, 2012, “A Phase 1, Randomized, Single-blind, Placebo-Controlled, Single Ascending Dose, Safety, Tolerability and Pharmacokinetics Study of ALN-TTR02 in Healthy Volunteers”). The results of these studies, both completed, were summarized by [109]. The two drugs were found to be safe and effective in reducing the TTR levels. In particular, ALN-TTR01, in which SNAPL are obtained by the Tekmira technique, and is administered to 32 patients with transthyretin amyloidosis, produced mild-to-moderate infusion-related reactions in 20.8% of patients, and at the highest dose of 1.0 mg/kg, a mean reduction at day seven of 38% for the TTR plasma level. ALN-TTR02, in which SNAPL are obtained by an LNP proprietary formulation [106], administered to 17 healthy volunteers, produced mild-to-moderate infusion-related reactions in 7.7% of patients, and at the highest dose of 0.3 mg/kg, reductions at day 28 of 56.6 to 67.1% for the TTR plasma level. Therefore, ALN-TTR02 was found to be more safe and effective than ALN-TTR01. Based on these results, Alnylam Pharmaceutics planned and carried out several more trials, a phase II (NCT 01617967, 2012, “A Phase 2, Open-Label, Multi-Dose, Dose Escalation Trial to Evaluate the Safety, Tolerability, Pharmacokinetics, and Pharmacodynamics of Intravenous Infusions of ALN-TTR02 in Patients With TTR Amyloidosis”) was completed with 29 patients enrolled; an extended phase II (NCT 01961921, 2013, “A Phase 2, Multicenter, Open-Label, Extension Study to Evaluate the Long-Term Safety, Clinical Activity, and Pharmacokinetics of ALN-TTR02 in Patients With Familial Amyloidotic Polyneuropathy Who Have Previously Received ALN-TTR02”) was also completed with 27 patients enrolled, followed by a phase III study (NCT 01960348, 2013, “APOLLO: A Phase 3 Multicenter, Multinational, Randomized, Double-blind, Placebo-controlled Study to Evaluate the Efficacy and Safety of ALN-TTR02 in Transthyretin (TTR)-Mediated Polyneuropathy (Familial Amyloidotic Polyneuropathy-FAP)”). In particular, in APOLLO study, which started in December 2013 and was completed in December 2018, 225 patients with hATTR amyloidosis polyneuropathy were enrolled and randomized 2:1 to receive 0.3 mg/kg Patisiran (name of the ALN-TTR02 formulation) or a placebo via intravenous infusion once every 3 weeks for 18 months. A consistent effect on the N-terminal prohormone of the brain natriuretic peptide at 18 months was observed in the overall APOLLO patients population (*n* = 225), showing good results also in the cardiac subpopulation (126 of 225 [56%]) patients treated with Patisiran compared with those treated with the placebo [110,111,112]. This trial represents the largest Phase 3 study of an RNAi strategy for the treatment of hATTR amyloidosis. Based on these results, an expansion study (NCT02939820 2016, “Expanded Access Protocol of Patisiran for Patients With Hereditary Transthyretin-Mediated Amyloidosis (hATTR Amyloidosis) With Polyneuropathy”) was carried out, providing further data on the long-term safety and efficacy of ALN-TTR02 in patients with hATTR amyloidosis, and thus on August 2018 ALN-TTR02 received a marketing license in Europe for stage 1 and 2 neuropathy in hATTR adult patients. 

Marketed as Onpattro™, Patisiran has become the first RNA interference lipid drug to win US Food and Drug Administration (FDA) approval, and is also the only specific medicinal treatment for this rare indication to gain approval in the USA. Two more studies of ALN-TTR02 are currently recruiting participants for a phase III clinical trials: NCT02510261, 2015, “A Multicenter, Open-Label, Extension Study to Evaluate the Long-term Safety and Efficacy of Patisiran in Patients With Familial Amyloidotic Polyneuropathy Who Have Completed a Prior Patisiran Clinical Study”—estimated enrollment 211 patients, starting date July 2015, estimated primary completion date Aug 2022; NCT03862807, 2019, “An Open-label Study to Evaluate Safety, Efficacy and Pharmacokinetics (PK) of Patisiran-LNP in Patients With Hereditary Transthyretin-mediated Amyloidosis (hATTR Amyloidosis) With Disease Progression Post-Orthotopic Liver Transplant”—estimated enrollment 20 participants, starting date Mar 2019, estimated primary completion date, Jan 2021.

4. siRNA-EphA2-DOPC

A neutral liposomes formulation loaded with a siRNA directed against the EPHA2 gene encoding for a tyrosine kinase which is overexpressed and implicated in tumor growth, was produced and tested in a preclinical study giving encouraging results, showing the good tolerability of nanoliposomal EphA2-targeted therapeutic (EPHARNA) in mice at different concentrations [113,114,115,116]. Therefore, a phase I dose-escalation clinical trial was started by the M.D. Anderson Cancer Center (NCT 01591356, 2012, “EphA2 Gene Targeting Using Neutral Liposomal Small Interfering RNA Delivery”). The study is currently recruiting participants, the estimated enrollment is of 40 subjects, the starting date was May 2012 and the estimated primary completion date is July 2020.

5. Atu027

AtuPLEX is a cationic LNP formulation produced by Silence Therapeutics containing siRNAs targeting the Cluster of Differentiation 31 (CD31) and the tyrosine kinase receptor for angiopoietins (TIE-2) mRNAs. The silencing of these endothelial cell–specific proteins can be a strategy to treat several kinds of advanced solid cancers. Obtaining a successful knockdown of the target genes into mouse vascular endothelium, an Atu027 formulation was produced using AtuPLEX technology to load siRNA, targeting the Protein Kinase N3 (PKN3), which is implicated in metastatic pancreatic tumor cell growth. Favorable preclinical data in animals [117,118] have led to a phase I clinical trial, based on Atu027 administered to patients with advanced solid tumors, by single and repeated intravenous infusion (NCT 00938574, 2009, “A Prospective, Open-label, Single Center, Dose Finding Phase I-study With Atu027 (an siRNA Formulation) in Subjects With Advanced Solid Cancer”). The study has been completed, the enrollment was of 34 subjects, the starting date was July 2009 and the study was concluded in April 2013. Atu027 was well tolerated up to dose levels of 0.336 mg/kg, showing low-grade toxicities (grade 1 or 2). The drug was safe in patients with advanced solid tumors, with 41% of patients having stable disease for at least 8 weeks [119,120]. In the light of these results, Silence Therapeutics began a new clinical trial, a phase I/II in which a combination of Atu027 and Gemcitabine was administered to patients with pancreatic carcinoma (APC) (NCT 01808638, 2013, “A phase Ib/IIa study of combination therapy with Gemcitabine and Atu027 in subjects with locally advanced or metastatic pancreatic adenocarcinoma”). The study has been completed, the enrollment was of 29 subjects with a starting date on Mar 2013 and a completion date on Jan 2016. The liposomal-formulated Atu027 in combination with Gemcitabine for the treatment of APC was safe and was well tolerated, a twice-weekly administration was better than a once-weekly regime, and the results are detailed in [121].

6. ND-L02-s0201

ND-L02-s0201 is a vitamin A–coupled cationic liposomal nanoparticle encapsulating an siRNA which inhibits the expression of Heat Shock Protein 47 (HSP47), a collagen-specific chaperone [122] involved in the fibrosis of liver and other organs. Vitamin A-moieties conjugated to the nanoparticles surface maximize the drug delivery to hepatic stellate cells. 

Preclinical data have suggested that disrupting collagen synthesis via HSP47 may reverse fibrosis. In particular, five treatments with the siRNA-vitamin A-coupled liposomes almost completely resolved liver fibrosis and prolonged survival in rats [123,124], thus a phase I study of ND-L02-s0201 was started by the Nitto Denko Corporation delivering the pharmaceutical by IV single-dose injection in healthy patients (NCT 01858935, 2013, “A Phase 1, Randomized, Double-Blind, Placebo-Controlled, Escalating Single Dose Study to Evaluate the Safety, Tolerability and Pharmacokinetics of ND-L02-s0201 Injection, a Vitamin A-Coupled Lipid Nanoparticle Containing siRNA Against HSP47, in Healthy Normal Subjects”). The study was completed, the enrollment was of 56 subjects with a starting date on May 2013 and a completion date on May 2017. 90 mg IV of ND-L02-s0201 for three weeks was well tolerated in healthy Japanese and non-Japanese adults enrolled, with no clinically meaningful differences or intolerability [123].

In the light of the encouraging results, the Nitto Denko Corporation carried out three more trials: A phase I to evaluate the safety and the tolerability of multiple doses of ND-L02-s0201 in subjects with moderate to extensive hepatic fibrosis (NCT02227459, 2014, “A Phase 1b/2, Open Label, Randomized, Repeat Dose, Dose Escalation Study to Evaluate the Safety, Tolerability, Biological Activity, and Pharmacokinetics of ND-L02-s0201 Injection, A Vitamin A-coupled Lipid Nanoparticle Containing siRNA Against HSP47, in Subjects With Moderate to Extensive Hepatic Fibrosis (METAVIR F3-4)”)—completed, 25 patients enrolled; an extended phase I to evaluate the safety, tolerability, and the effects of two ND-L02-s0201 (NCT03241264, 2017, “A Phase 1, Open-Label, Randomized-Sequence, Single-Crossover, Bridging Study to Evaluate the Single-Dose Pharmacokinetics, Safety, and Tolerability of Two ND-L02-s0201 Formulations, Frozen Versus Lyophilized, Administered by Intravenous Infusion to Healthy Male and Female Subjects“)—completed, 12 patients enrolled; a phase II study (NCT03538301, 2018, “A Phase 2, Randomized, Double-Blind, Placebo-Controlled Study to Evaluate the Safety, Tolerability, Biological Activity, and PK of ND-L02-s0201 in Subjects With Idiopathic Pulmonary Fibrosis (IPF)“)—currently recruiting participants, with an estimated enrollment of 120 participants. The initial date was May 2018, with an estimated primary completion date in Mar 2020.

7. TKM-ApoB

TKM-ApoB is a systemically-delivered RNAi therapeutic based on SNALP carrying an siRNA against the ApoB (Apolipoprotein B) mRNA in order to downregulate the protein which facilitates the uptake of LDL into various cell types and tissues, reducing the LDL cholesterol levels in hypercholesterolemia patients. In the light of the positive preclinical results [100] a phase I clinical trial has been initiated (NCT 00927459, 2009, “Study to Evaluate the Safety, Tolerability, Pharmacokinetics (PK), and Pharmacodynamics (PD) of Liposomal siRNA in Subjects With High Cholesterol”). The enrollment was of 23 subjects with a starting date on June 2009, but the study has been terminated [125]. Of the 23 subjects, 17 received treatment with a single dose IV infusion of the TKM-ApoB at seven different dosing levels, the other six subjects received the placebo control. The drug was well tolerated, with no evidence of liver toxicity, except for one of the two subjects treated with the highest drug dosage, who, although reporting an ApoB protein and LDL cholesterol reduction, has also reported symptoms of immune system stimulation [12]. Tekmira has declared it was not satisfied with the performance of its current TKM-ApoB LNP formulation, and thus they will be going to focus on selecting an alternative formulation with improved nanoparticle carriers and siRNA chemistry to recommence a clinical trial for the ApoB siRNA therapy.

8. TKM-080301

The TKM-080301 is a SNALP formulation of a siRNA against Polo-Like Kinase 1 (PLK1), a serine/threonine kinase that regulates key aspects of cell cycle progression and mitosis and is highly expressed in malignant cells. In preclinical models the anti-tumor activity of PLK1 inhibition through RNA interference have been demonstrated [126,127,128], thus several clinical trials based on the pharmaceutical TKM-080301 were carried out by Tekmira Pharmaceuticals. 

At first, the feasibility of administering TKM-080301 via Hepatic Arterial Infusion (HAI) and its pharmacokinetics and pharmacodynamics in patients with unresectable primary liver cancer or liver metastases has been evaluated in a dose-escalation study (NCT 01437007, 2011, “A Phase 1 Dose Escalation Study of Hepatic Intra-Arterial Administration of TKM-080301 (Lipid Nanoparticles Containing siRNA Against the PLK1 Gene Product) in Patients With Colorectal, Pancreas, Gastric, Breast, Ovarian and Esophageal Cancers With Hepatic Metastases”). The study started in Aug 2011 and was completed in June 2012. Subsequently, to determine the safety and tolerability of TKM-080301 in adult patients with solid tumors or lymphomas that are refractory to standard therapy, or for whom there is no standard therapy, a phase I/II TKM-PLK1 clinical trial was carried out (NCT 01262235, 2010, “A Phase 1/2 Dose Escalation Study to Determine the Safety, Pharmacokinetics, and Pharmacodynamics of Intravenous TKM-080301 in Patients With Advanced Solid Tumors”). The study was completed, the enrollment was of 68 subjects, the starting date was Dec 2010 and the primary completion date was July 2015. Results of NCT 01437007 and NCT 01262235, reported in [129,130], indicate that TKM-080301 was generally well-tolerated by the majority of patients showing a preliminary antitumor efficacy, supporting PLK1 as a therapeutic target. Finally, to test the safety and tolerability of TKM-080301 in subjects with advanced hepatocellular carcinoma and to find the maximum tolerated dose (MTD) providing a preliminary assessment of anti-tumor activity of TKM-080301, an extended phase I/II TKM-PLK1 clinical trial for HCC was done (NCT 02191878, 2014, “Open-Label, Multi-Center, Phase 1, Dose Escalation Study With Phase 2 Expansion Cohort to Determine the Safety, Pharmacokinetics and Preliminary Anti-Tumor Activity of Intravenous TKM-080301 in Subjects With Advanced Hepatocellular Carcinoma”). The study was completed, the enrollment was of 43 participants, the starting date was Jun 2014 and the primary completion date was May 2016. TKM-080301 was generally well tolerated, with a starting dose of 0.3 mg/kg and MTD of 0.6 mg/kg. Four patients did not have any evaluable post baseline scan. Of the other 39 subjects who had received at least 0.3 mg/kg, 18 subjects (46.2%) had stable disease and eight subjects (23.1%) had a partial response. The median survival for the whole study population was 7.5 months. Results are reported in [131].

9. TKM-100201

TKM-100201 is a small interfering RNA lipid nanoparticle product which has been developed for the treatment of the Ebola Virus Disease (EVD), an infection characterized by an immune suppression and a systemic inflammatory response that causes the impairment of the vascular, coagulation and immune systems, leading to multiorgan failure [132]. The formulation is a mixture of three siRNAs that target the L, VP24, and VP35 proteins of the Zaire Ebola Virus strain based on SNALP technology. In preclinical studies the pharmaceutical efficiently provided a post-exposure protection in animal models [98,99,133], therefore, Tekmira Pharmaceutical initiated a phase I clinical trial based on IV infusion with dose-escalation (NCT01518881, 2011, “A Placebo-Controlled, Single-Blind, Single-Ascending Dose Study With Additional Multiple-Ascending Dose Cohorts to Evaluate the Safety, Tolerability and Pharmacokinetics of TKM-100201 in Healthy Human Volunteers”). The study started in Jan 2012, but was terminated because Tekmira Pharmaceutical has decided to reformulate the product, which presented a dangerous EBOV-induced inflammatory response [134].

10. TKM-100802

As a consequence of the previous clinical trial, Tekmira Pharmaceutical modified the drug TKM-100201, toward TKM-100802, a lyophilized formulation with an increased therapeutic index. The LNP and the pool of siRNAs remained unchanged, but the new formulation achieved 100% survival (6 primates over 6), using a 4-fold lower dose (0.5 mg/kg/dose), and a significant survival advantage (4 primates over 6) even at the 10-fold lower dose (0.2 mg/kg/dose) [135]. The new drug has undergone a phase I trial, administered by IV infusion to healthy patients, with dose-escalation (NCT02041715, 2014, “A Placebo-Controlled, Single-Blind, Single-Ascending Dose Study With Additional Multiple-Ascending Dose Cohorts to Evaluate the Safety, Tolerability, and Pharmacokinetics of TKM-100802 in Healthy Human Volunteers”). 

The Single Ascending Dose (SAD) part of the study has interested 14 patients, and Adverse Effects (AEs) have been observed at the highest dose (0.5 mg/kg/dose); thus also this study was terminated in Aug 2015 due to safety concerns arising from the development of flu-like symptoms in treated individuals, which were ultimately linked to cytokine release triggered by the action of the siRNA [133,136].

A TKM-130803 siRNA LNP-enhanced formulation in which the siRNA component has been adapted by two nucleotide substitutions in the VP35 siRNA and a single nucleotide substitution in the L-polymerase siRNA to ensure specificity to the West African Makona variant of Zaire Ebola Virus, has been developed for EVD therapy [137]. However the new product, administered at a dose of 0.3 mg/kg/d by intravenous infusion to adult patients with severe EVD, was not shown to improve survival when compared to historic controls. On 14 patients enrolled, 11 died and 3 survived, thus the trial was halted, and is not reported in *ClinicalTrials.gov* [138].

To date, the development of new approaches to fight EBOV are in progress, and a genome-wide siRNA screen to identify novel pro- and antiviral factors against the Ebola Virus life cycle is being performed [139].

11. DCR-MYC

DCR-MYC is a lipid nanoparticle-based formulation consisting of an siRNA directed against the mRNA of the proto-oncogene c-Myc, involved in cellular proliferation, differentiation and apoptosis, and overexpressed in a variety of cancers. The inhibition of c-Myc translation has a potential antineoplastic activity as demonstrated in preclinical studies [140]. Therefore, a phase I study of DCR-MYC in patients with solid tumors and hematological malignancies was started by Dicerna Pharmaceuticals, delivering the drug by IV infusion (NCT 02110563, 2014, “Phase I, Multicenter, Dose Escalation Study of DCR-MYC in Patients With Solid Tumors, Multiple Myeloma, or Lymphoma”). This study, started in Apr 2014, was immediately followed by the subsequent phase I/II clinical trial (NCT 02314052, 2014, “Phase 1b/2, Multicenter, Dose Escalation Trial to Determine the Safety, Tolerance, Maximum Tolerated Dose and Recommended Phase 2 Dose of DCR-MYC, a Lipid Nanoparticle (LNP)-Formulated Small Inhibitory RNA (siRNA) Oligonucleotide Targeting MYC, in Patients With Hepatocellular Carcinoma (HCC)”). Unfortunately, despite showing promising initial clinical and metabolic responses across various dose levels [141], DCR-MYC clinical trials were terminated for “Sponsor decision” because preliminary results did not meet the Company’s expectations for further developments.

12. ARB-001467

The ARB-001467 is an RNA interference agent comprised of three siRNAs delivered using lipid nanoparticle technology, in particular liposomes, targeting the Hepatitis B Virus (HBV) RNA, and inhibiting the production of all HBV proteins, reducing the cccDNA content [142]. Preclinical studies demonstrated that a single dose of ARB-001467 can reduce HBsAg in a chimeric mouse model while ARB-001467 multiple injections led to a 90% reduction of HBsAg levels and a 50% reduction of cccDNA within 28 days of treatment in chimpanzees [143]. Based on these results, the Arbutus Biopharma corporation carried out a phase II clinical trial (NCT02631096, 2015, “A Phase 2a Single-Blind, Randomized, Placebo-Controlled Study Evaluating the Safety, Anti-Viral Activity, and Pharmacokinetics of ARB-001467 in Non Cirrhotic, HBeAg Negative and Positive Subjects With Chronic HBV Infection Receiving Nucleos(t)Ide Analogue Therapy”). The study was completed, the enrollment was of 36 participants, the starting date was Dec 2015 and the completion date was May 2018. Subjects were enrolled in three cohorts: Cohort 1, HBeAg-negative (0.2 mg/kg); Cohort 2, HBeAg-negative (0.4 mg/kg); Cohort 3, HBeAg-positive (0.4 mg/kg) and ARB-001467 was given as an intravenous injection over two hours, monthly, for three months. The treatment was generally well tolerated, and all subjects receiving ARB-001467 experienced a reduction in HBsAg from the baseline; greater HBsAg reductions were observed with more frequent dosing (bi-weekly), and at the higher dose (0.4 mg/kg) [144].

### Challenges and Limitations of Lipid-Based siRNA Delivery Systems

Good biocompatibility, biodegradability, low toxicity, structural variability, easiness of largescale production and the possibility of both hydrophilic and lipophilic drugs incorporation are all beneficial properties that make lipid-based systems advanced NABDs delivery vectors with respect to other carriers [145].

In fact, vectors based on polymers such as chitosan, cyclodextrin polyethyleneimine, poly lactic-*co*-glycolic acid, dendrimers (i.e., polycationic dendrimers as polyamidoamine (PAMAM) and polypropylenimine (PPI)), although being excellent candidates for the delivery of NABDs, constitute only a small number of gene vectors entered in clinical trials (i.e., siG12D LODER (Local Drug EluteR) for Pancreatic Cancer [146]) with respect to the lipid-based ones. Also considering carriers based on peptides, proteins or the so-called “next-generation nanoparticles” or rather viral capsids, as noninfectious protein-based nanoparticles, termed virus-like particles (VLPs) [147], the similarity of lipid based systems with biological membranes, with all the consequent advantages such as a facilitated uptake in the cells and a greater biocompatibility, makes them unique carriers increasingly promising in gene therapy.

Also siRNA chemical modifications have been rigorously investigated with the goal of prolonged half-life, increased cellular uptake and targeted delivery if the nucleic acid is conjugated with proteins, aptamers, cholesterol and its derivatives, cell penetrating peptides (CPPs) and carbohydrates. For instance, the delivery of RNAi therapeutics to liver hepatocytes by subcutaneous administration has been achieved by Alnylam Pharmaceuticals using the GalNAc-siRNA conjugate delivery platform. In this approach, the siRNAs are conjugated with multivalent N-acetylgalactosamine (GalNAc) residues that are recognized by the asialoglycoprotein receptor (ASGPR) expressed on human hepatocyte cell surfaces with the consequent endocytosis. GalNAc-siRNA conjugates are reactive in numerous animal models, and thus are significantly promising for targeting disease-causing genes produced in liver [148].

Anyhow, in recent years the attention of the scientific community has focused on the development of vectors for NABDs delivery and several companies, including Tekmira, Alnylam, Silence Therapeutics, and others, have introduced siRNA nanoparticle products in either the preclinical or clinical phases [13]. In particular, the use of lipid carriers systems for NABDs delivery has given excellent feedback in clinical applications, while the recent approval of ALN-TTR02 for marketing this product is certainly the proof, but it is not the only example. The favorable features of these vectors are also reflected in all those products that have successfully passed one or more phases of clinical trials, i.e., showing to be safe and well-tolerated, able to improve the pharmacokinetics and pharmacodynamics of siRNAs. However, despite the use of new synthesized lipids and technologies such as SNALP and other LNP-based products, toxicity and immunogenicity still represent a great hindrance for these novel class of therapeutics [149].

Moreover the successful use of a type of carrier system for a certain disease does not guarantee its efficiency for the treatment of another illness, i.e., the phase I clinical trial of TKM-ApoB formulation based on LNP technology, and containing an siRNA targeting ApoB to be used for the treatment of hypercholesterolemia, was terminated due to patients’ immune systems stimulation, as well as another Tekmira Pharmaceutical product developed to combat the Ebola Virus (TKM-100802). On the contrary, the same LNP technology, in particular the SNALP subfamily, was successfully used for the cure of TransThyRetin (TTR)-mediated amyloidosis through the development of ALN-TTR02 or for the treatment of solid tumors, this through ALN-VSP02 formulation, which has passed the phase I of clinical trials.

Finally, the problem represented by the high manufacturing cost of these therapeutic products must be considered, i.e., Patisiran treatment is currently priced at $450,000 per patient per year.

## 5. Conclusions

The wide range of diseases that can be treated by NABDs which are being evaluated in clinical trials, ranging from tumors to viral infections, highlight the great therapeutic potential of this new class of biological therapeutics, whose limit is determined by the impossibility of being administered in their naked form. To date, engineered lipid delivery systems, as cationic liposomes used in the SNALP of Tekmira Pharmaceuticals, represent the approach of choice for a concrete NABDs delivery; however, although the technology has undergone rapid developments, becoming a reality with the marketing of the first RNA- lipid-based drug carrier, some barriers still need to be overcome.

Currently, the lack of a rapid, reliable and scalable technique, reflecting in NABDs-liposomes the high production cost for minimum amounts of product, and the impossibility of having a meticulous control over the possible biological responses following their administration, i.e., toxicity and immunogenicity, represent the major limits, thus their overcoming is the challenge to face in order to get, in the near future, more powerful alternatives to the common drugs for the cure of several disabling diseases.

## Figures and Tables

**Table 1:** The siRNA-lipid delivery systems used in clinical trials.

#	Drug	Target	Disease	Company	[104]		
					Identifier	Phase	Status
1.	ALN-VSP02	KSP and VEGF	Solid tumors	Alnylam Pharmaceuticals	NCT 00882180	I	Completed
					NCT 01158079	I	Completed
2.	ALN-PCS02	PCSK9	Hypercholesterolemia	Alnylam Pharmaceuticals	NCT 01437059	I	Completed
3.	ALN-TTR01	TTR	TransThyRetin (TTR)-mediated amyloidosis	Alnylam Pharmaceuticals	NCT 01148953	I	Completed
	ALN-TTR02				NCT 01559077	I	Completed
					NCT 01617967	II	Completed
					NCT 01961921	II	Completed
					NCT 01960348	III	Completed
					NCT02939820	Approved for marketing	
					NCT02510261	III	Recruiting
					NCT03862807	III	Recruiting
4.	siRNA-EphA2-DOPC	EPHA2	Advanced cancers	M.D. Anderson Cancer Center	NCT 01591356	I	Recruiting
5.	Atu027	PKN3	Advanced solid cancers	Silence Therapeutics	NCT 00938574	I	Completed
					NCT 01808638	I/II	Completed
6.	ND-L02-s0201	HSP47	Fibrosis	Nitto Denko Corporation	NCT 01858935	I	Completed
					NCT02227459	I	Completed
					NCT03241264	I	Completed
					NCT03538301	II	Recruiting
7.	TKM-ApoB	Apo B	Hypercholesterolemia	Tekmira Pharmaceuticals	NCT 00927459	I	Terminated
8.	TKM-080301	PLK1	Cancer (Polo-Like-Kinase 1)	Tekmira Pharmaceuticals	NCT 01437007	I	Completed
					NCT 01262235	I/II	Completed
					NCT 02191878	I/II	Completed
9.	TKM-100201	VP24, VP35, l-polymerase	Ebola Virus Infection	Tekmira Pharmaceuticals	NCT 01518881	I	Terminated
10.	TKM-100802	VP24, VP35, l-polymerase	Ebola Virus Infection	Tekmira Pharmaceuticals	NCT 02041715	I	Terminated
11.	DCR-MYC	MYC	Solid Tumors Multiple Myeloma Non-Hodgkins Lymphoma	Dicerna Pharmaceuticals	NCT 02110563	I	Terminated
			Hepatocellular Carcinoma		NCT02314052	I/II	Terminated
12.	ARB-001467	HBV proteins	Hepatitis B, Chronic	Arbutus Biopharma	NCT02631096	II	Completed
